# Radiological evaluation of the eighth thoracic vertebra rotation in the pectus excavatum

**DOI:** 10.1186/1748-7161-10-S1-P19

**Published:** 2015-01-19

**Authors:** Ryszard Tomaszewski, Lumila Machala, Artur Gap

**Affiliations:** 1 Upper Silesian Child Health Centre, Katowice, Poland

## Objective

The main objective was to find the difference between the rotation of the eighth thoracic vertebra in symmetric and dissymmetric pectus excavatum in children.

## Material and methods

An analysis of pre-operational 82 CT in children with the pectus excavatum deformity was made. Patients were divided into two groups depending on the chest deformation ; the first one consisted of patients with symmetric pectus excavatum, there were 48 patients (9 girls, 39 boys), the mean age was 12,8 years (4-16), the average Haller index was 3,6 (2,2-7,1); in the second group with dissymmetric pectus excavatum there were 35 patients (8 girls, 27 boys), the mean age was 11,8 years (7-17), the average Haller index was 2,9 (2,4-5,8).

## Results

In the first group the rotation of the eighth thoracic vertebra was found in 60,4% (29 patients), with mean rotation angle of 6,29 (2,6-32), the average Haller index was 3,7 (2,2-7,1), In the second group there were 45,7 % (16 patients) with the mean rotation angle of 4,72 (2,4-10,5), the average Haller index was 5(2,4-4,9).

## Conclusion

The rotation of the eighth thoracic vertebra is significantly more common in symmetric pectus excavatum in children than in dissymmetric deformity.

